# Weekly Iron Folate Supplementation in Adolescent Girls – An Effective Nutritional Measure for the Management of Iron Deficiency Anaemia

**DOI:** 10.5539/gjhs.v5n3p188

**Published:** 2013-03-20

**Authors:** Mohan Joshi, Raghvendra Gumashta

**Affiliations:** 1 Department of Community Medicine, NKP Salve Institute of Medical Sciences and Research Centre, Nagpur, Maharashtra, India

**Keywords:** anaemia, compliance, iron folate, weekly regime, daily regime, ADR: Adverse Drug Reaction, ASHA: Accredited Social Health Activist, Hb: Haemoglobin, DIFS: Daily Iron Folic Acid Supplementation, IDA: Iron Deficiency Anaemia, IFA: Iron and Folic Acid/Iron Folate, MRP: Maximum Retail Price, MMR: Maternal Mortality Rate, NIN: National Institute of Nutrition, SE: Side Effects, SES: Socio Economic Status, ‘UHTC’: Urban Health and Training Centre, WIFS: Weekly Iron Folic Acid Supplementation

## Abstract

**Introduction:**

Nutritional anaemia in India is common morbidity seen in late adolescent and young female population. There are many conflicting opinions regarding dosage of iron folic acid supplementation for managing this simple nutritional deficiency disorder. Hence, this ‘Randomized Controlled Trial’ was undertaken in adolescent girls suffering from Iron Deficiency Anaemia visiting ‘Urban Health and Training Centre’ situated in urban slum area. The aim of this study was to assess the (a) Impact of weekly iron folic acid supplementation in comparison with daily iron supplementation for the management of Iron Deficiency Anaemia in adolescent girls visiting ‘Urban Health and Training Centre’; (b) Adverse drug reaction profile in ‘Weekly Iron Folic Acid Supplementation’ and ‘Daily Iron Folic Acid Supplementation’ regimes; (c) Compliance profile for ‘Weekly Iron Folic Acid Supplementation’ and ‘Daily Iron Folic Acid Supplementation’ regimes in adolescent girls.

**Methods and Material:**

Randomized controlled trial was conducted in adolescent girls visiting ‘Urban Health and Training Centre’ during the study period June, 2011 to October, 2012. The 120 anaemic (Haemoglobin < 12 gm%) adolescent girls (10-19 years) were distributed randomly by block randomization in two groups; one receiving daily Iron and Folic Acid supplementation and in other group receiving weekly Iron and Folic Acid supplementation for 3 months. All the study subjects were given de-worming (Albendazole 400 mg) and required health education separately. Both the groups were monitored for Haemoglobin estimation, compliance and adverse drug reactions, if any. Open-Epi Statistical Software was used for data analysis.

**Results:**

The mean age of study subjects in ‘Daily Iron and Folic Acid Supplementation’ and ‘Weekly Iron and Folic Acid Supplementation’ group was 13.48 and 13.55 years respectively. Their mean pre intervention Haemoglobin was 10.1±1.1 gm/dl and 10.4±1.1 gm/dl respectively. The mean rise in Haemoglobin after lean period of 1 month in respective groups was almost equal i.e. 1.0±0.7 gm/dl and 1.0±0.8 gm/dl. Adverse Drug Reactions were 8.3% in weekly regime as compared to 13.35% in daily regime, abdominal pain being the commonest adverse drug reaction seen. The compliance calculated as mean of unconsumed ‘Iron and Folic Acid’ tablets was 6.1±10.98 in ‘Daily Iron Folic Acid Supplementation’ group, while it was 1.3±3.15 in ‘Weekly Iron Folic Acid Supplementation’ group (p=0.0012), making weekly regime more promising than daily regime with better treatment compliance.

**Conclusions:**

Weekly supplementation of ‘Iron and Folic Acid’ in ‘Iron Deficiency Anaemia’ patients is as good as daily supplementation with added benefits of less adverse reactions and better compliance.

## 1. Introduction

World interest in adolescent health issues has grown dramatically beginning with the international year of youth in 1985 and the world health assembly sessions held thereafter (Kaur, Deshmukh, & Garg, 2006). Among adolescents, girls constitute a vulnerable group, particularly in developing countries, where they are traditionally married at an early age and exposed to a greater risk of reproductive morbidity and mortality. Among the adolescent girls the prevalence of anaemia is disproportionately high in developing countries, due to poverty, inadequate diet, worm infestations, and frequent attacks of malaria in presence of poor access of health services. It is accepted fact that nutritional anaemia in adolescent girls attributes to high MMR, high incidence of low birth weight babies, high perinatal mortality, fetal wastage and consequent high fertility rates. There is increasing evidence that control of anaemia in pregnant women may be more easily achieved if satisfactory iron status can be achieved in adolescent age (WHO, 1999).

Anaemia is a worldwide problem most commonly due to wide spread nutritional deficiencies. In adolescent girls after onset of menarche, anaemia may impair immune system response which at times predisposes this vulnerable group for increased morbidity and consequent poor health. The prevalence of anaemia in different parts of this country varies from 34.5% to 65% depending on availability of qualitatively and quantitatively adequate food in their respective families (Rawat, Garg, & Singh, 2001). The girls not attending schools belong to disadvantaged section of society and contribute significantly to domestic and economic activities, which at times jeopardize their health and have high prevalence of nutritional anaemia amongst them.

The idea of weekly iron supplementation was conceived as a preventive rather than a therapeutic measure for iron deficiency and its progression to anaemia. This preventive approach considers the capacity of fresh intestinal cells, to absorb iron and deliver it to transferrin in response to internal signals reflecting total body iron status and homeostatic need. The human intestinal mucosal turnover occurs every 5 to 6 days and preventing iron only to new mucosal cells for optimum absorption seemed to be attractive option for prevention of iron deficiency (Viteri & Berger, 2005). The model tested in rats demonstrated that iron assimilation declined more slowly and was more than 2.5 times more efficient than when same dose was administrated daily (Viteri, Liu, Martin, & Tolomei, 1995). In the last decade, the overwhelming majority of studies in different parts of the world with population of different ages and sex have shown that the weekly approach is efficacious and effective as daily approach. There are certain conflicting reports in this regard because these studies were short term and conducted in essentially iron normal population (Agarwal, Gomber, Bisht, & Som, 2003). Long term absorption in iron deficient population demonstrates a more efficient, sustained absorption from weekly supplementation.

Hence, we planned IFA supplementation study only in iron deficient study subjects i.e. those suffering from anaemia (Hb< 12 gm%). There are many studies conducted in general population or even in adolescent girls from rural, urban or tribal region but there are very few studies in anemic adolescent girl population. Recently it has been learned that GOI has also recommended WIFS as intervention for prevention and control of IDA in adolescent school children. This study is planned to provide a scientific basis for these interventions. Hence the need of present study is justified.

The aim of the study was to assess the (a) Impact of ‘weekly iron folate supplementation’ in comparison with ‘daily iron supplementation’ for the management of Iron Deficiency Anaemia in adolescent girls visiting ‘Urban Health and Training Centre’; (b) Adverse drug reaction profile in ‘Weekly Iron Folic Acid Supplementation’ and ‘Daily Iron Folic Acid Supplementation’ regimes; (c) Compliance profile for ‘Weekly Iron Folic Acid Supplementation’ and ‘Daily Iron Folic Acid Supplementation’ regimes in adolescent girls.

## 2. Material and Methods

After obtaining the permission of Institutional Ethics Committee, this randomized controlled trial was conducted among all the adolescent girls visiting ‘Urban Health and Training Centre’ during the study period June, 2011 to October, 2012 who were interviewed as per pretested proforma. This activity was preceded by one day ‘hands on training’ related with the study requirements to Medical Social Worker and Laboratory Technician of ‘Urban Health and Training Centre’ for ensuring strict adherence to study protocol. All adolescent girls visiting ‘Urban Health and Training Centre’ were investigated for the hemoglobin estimation by Sahli’s Hemoglobinometer. Those adolescent girls with Hb < 12 gmand consenting for this study were included in present study, and were given de-worming dose (albendazole 400 mg stat and after 15 days) and related health education in separate sessions. Exclusion criteria set for this study was (a) Pregnant teenage mothers; (b) chronic conditions like TB/Renal/Menorrhagia/Metrorrhagia; (c) history of acute illness like malaria in recent time (< 3 months); and (d) girls with known diagnosed morbidities like Sickle Cell Anaemia.

The 120 anaemic (Haemoglobin < 12 gm%) adolescent girls (10-19 years) were distributed randomly in two groups; one receiving daily IFA supplementation and other group receiving weekly IFA supplementation for 3 months. There were two groups of participants (Group A – ‘Daily IFA Supplementation Group’ and Group B – ‘Weekly IFA Supplementation Group’) comprising of 60 girls in each group. Randomization was done by using computer generated block randomization, four in each block. Every time when a adolescent girl with Hb< 12 gm/dl was selected for inclusion in this study, she was allotted randomly in either group by laboratory technician using block randomization, till required quota for 60 subjects in each group was completed. The iron folate supplementation was done by providing Capsule Succiron (hard gelatine capsule containing Ferrous Fumerate IP 300 mg; Vitamin B12, in gelatine, 15 mcg; Folic Acid IP 1.5 mg), manufactured by EISEN Pharmaceuticals Co. Pvt. Ltd of Pune (Mfg. Lic. No. 106) costing MRP 22.00 Rs. per 10 capsules. These were made available for free distribution among the study subjects by the said pharmaceutical company without any conflict of interest. Every girl with Hb< 12 gm % was given daily or weekly iron folate supplementation as per their randomized group allocation for a period of 3 months. The Health Education session for both the groups were organized separately, and included briefing about natural sources of Iron, Vitamin C, Folic Acid and facilitators and inhibitors of Iron absorption. The compliance of the designated schedule was checked from time to time through home visits and interview method by Field Worker/Supervisor/Investigators. Empty cartons were also collected from them for assessment of the IFA intake. After the lag period of one month post intervention, all the study subjects were interviewed for compliance to the interventions and adverse drug reaction profile. The post intervention Haemoglobin estimation was also be done. The results were analyzed thereafter by Open Epi Statistical Software and matched pair t-test was applied for comparison of findings among study subjects randomized into two groups as per the protocol. Those needing referral were referred to tertiary care hospital for further management.

## 3. Results

Overall prevalence of anaemia was brought down by 25% in ‘Daily Iron Folic Acid Supplementation’ group after the supplementation for 3 months while it was brought down by 31.67% in weekly supplementation group. The prevalence of moderate anaemia was brought down from 36.67% to 10% in ‘Daily Iron Folic Acid Supplementation’ group while for weekly regime it was brought down from 26.67% to 6.6% (Table 1). The prevalence of mild anaemia had remained unchanged pre and post intervention in both daily and weekly regimes, which may have been due to shifting of certain subjects from moderately anemic group to mildly anemic group in both the regimes. As there was no study subject with severe anaemia in weekly supplementation group and only one subject in daily supplementation group, the results were not be compared during analysis.

**Table 1:** Comparison of anaemia status before and after intervention among daily IFA supplementation (DIFS) and weekly IFA supplementation (WIFS) groups

Hb (gm%)	No. of adolescents						Inter group ‘p value’ by matched pair t test of ‘after intervention’ results among both groups: (b) and (d)

	Daily IFA Supplementation(n=60)			Weekly IFA Supplementation(n=60)			

	Before intervention(a)	After intervention(b)	P value	Before intervention(c)	After intervention(d)	p value	
**>12 (Normal)**	00	15(25%)		00	19(31.67%)		0.001
**10-11.9 (Mild Anaemia)**	37(61.67%)	39(65%)	< 0.001	44(73.33%)	37(61.67%)	< 0.001	
**7-9.9 (Moderate Anaemia)**	22(36.67%)	06(10%)		16(26.67%)	04(6.66%)		
**< 7 (Severe Anaemia)**	01(1.66%)	00		00	00		
**Total**	60 (100%)	60 (100%)		60 (100%)	60 (100%)		

The rise of mean hemoglobin level in daily IFA supplementation was 1.04+ 0.7 gm/dl while it was 1.0+ 0.8 gm/dl in weekly IFA supplementation group. When both these group results were compared by using two samples independent t test with 2 side CI 95%, then p value for rise of mean Haemoglobin was 0.53. Though this value is statistically non-significant, it also makes a point that both the groups are similar in efficacy of regimes. When these regimes are analyzed for effectiveness, both daily and weekly regimes showed positive significant results (p<0.001) (Table 2). The results are really encouraging for weekly IFA supplementations as good alternative regimes for prevention and control of anaemia in adolescent girls.

**Table 2:** Comparison of mean Hb level before and after intervention among daily IFA supplementation and weekly IFA supplementation groups (By “Two Sample Independent t Test” with 2 side C.I. 95%)

Hb (gm%)	Daily IFA Supplementation Group(n=60)				Weekly IFA Supplementation Group(n=60)				Difference of mean rise of Hb (gm%) DIFAS Vs. WIFAS	p value for ‘rise of mean Hb (gm%)’ among DIFAS and WIFAS Groups

	Mean Hb (gm%)			Rise of mean Hb (gm%)	Mean Hb (gm%)			Rise of mean Hb (gm%)		
	
	*B.I.	*A.I.	p value		*B.I.	*A.I.	p value			
10-11.9(Mild Anaemia)	10.8+0.5	11.6+ 0.8	< 0.001	+0.8+ 0.7	11.0+ 0.6	11.7+ 0.7	< 0.001	+0.6+ 0.6	+ 0.2	
7-9.9(Moderate Anaemia)	9.25+0.52	10.61+ 0.66	< 0.001	+1.37+ 0.67	8.98+0.97	10.67+ 0.97	< 0.001	+1.68+ 0.82	- 0.31	
< 7 (Severe Anaemia)	5.6	7.4	-	1.8	NA	NA	-	NA	NA	
**Total**	10.1+1.1	11.2+ 1.0	< 0.001	**1.0+ 0.7**	10.4+1.1	11.4+ 0.9	< 0.001	**1.0+ 0.8**	0.0	0.53

* B.I.: Before Intervention; A.I.: After Intervention; NA: Not Applicable

There was significant post intervention improvement in anaemia status among all the study subjects who were moderately anemic and prevalence of moderate anaemia was reduced from 31.66% to 8.33% (i.e. the reduction of 23.33%); while for the study subjects who were mildly anemic pre-intervention had marginal change of 4.2% in post intervention anaemia status. This could be due to post intervention shifting of certain moderately anemic subjects to mild anemic status and further 28.33% of study subjects reported normal Hemoglobin status (Hb>12 gm/dl) post intervention (Table 1).

When data was analyzed for ADR in both groups (Table 3), abdominal pain was the common ADR noted in 11.6% study subjects. The total of 8.3% study subjects has ADR in weekly IFA group while for daily IFA group about 13.3% study subjects had ADR, which is marginally higher than weekly IFA group. The abdominal pain which is commonest ADR seen in 6.6% of daily IFA group, where constipation, nausea and skin rash were also other ADR seen in them. So, it can be summarized that weekly IFA supplementation is better tolerated than daily IFA among the study subjects.

**Table 3:** Adverse drug reactions in daily IFA supplementation group and weekly IFA supplementation group

Adverse Drug Reactions(ADR)	Daily IFA Supplementation Group(n=60)	Weekly IFA Supplementation Group(n=60)
Constipation	2	0
Nausea	1	0
Abdominal Pain	4	3
Rash	1	0
Others	0	2
**Total**	**8**	**5**
**% study subjects having ADR**	**13.3**	**8.3**

When the data was analyzed to study the pattern of compliance given to these two different regimes (Table 4), it is seen that mean of unconsumed IFA was 6.1 ± 10.98 in daily IFA group while it was 1.3 ± 3.15 in weekly IFA group. Hence it could be summarized that weekly IFA supplementation had higher compliance as compared to daily IFA supplementation. The application of ‘t test’ for the said data inferred p value to be 0.0012, which shows significant difference in the mean of unconsumed IFA tablets between these two groups.

**Table 4:** Observed IFA intake compliance among the two groups

	Daily IFA Supplementation Group (n=60)	Weekly IFA Supplementation Group (n=60)	p value
Total IFA tablets given	5400	780	
Total unconsumed IFA tablets	366	78	
No. of IFA tablets provided to each participant after counselling session	90	13	
Mean of unconsumed IFA tablets	6.1 ± 10.98	1.3 ± 3.15	0.0012

## 4. Discussion

The results from present study carried out in adolescent girls selected from urban slum population of metropolitan city of Nagpur clearly provide basis for weekly IFA supplementation in adolescent girls. The mean rise of Haemoglobin perentage in daily and weekly IFA supplementation group is 1.04 ± 0.7 gm/dl and 0.95 ± 0.8 gm/dl within a span of 3 months. The efficacy of IFA supplementation on weekly basis is comparable to daily iron folic acid supplementation. There is a significant post intervention improvement in anaemia status, particularly among the subjects having moderate anaemia, with reduction in anaemia prevalence to the tune of 23%.

Kotecha et al. (2009) in their effort to institutionalize once a week IFA supplementation covered 69790 girls from 426 schools in Vadodara district of Gujarat and reported reduction in anaemia prevalence by 21.5 %, from 74.7% to 53.2% (p<0.05) and also showed corresponding improvement in serum ferritin values. They also reported the reduction of 68% in prevalence of severe anaemia while it was 51% and 22% in moderate and mild anaemia respectively. The overall reduction anaemia was about 28.8% at 12 gm/dl cut off point after weekly IFA supplementation for 12 months. The mean rise of hemoglobin was 0.64 gm/dl with regional differences seen maximum in rural area followed by urban area and relatively least rise was seen in tribal area. The results of the present study are comparable with this study. Results shown by Casey et al. (2009) showed that free, universal WIFS with regular deworming was associated with a reduced prevalence and severity of anaemia and hookworm infection in non-pregnant women over a 12-month period. According to them, countries with high rates of anaemia in women should urgently consider WIFS and regular deworming as interim measures until sustainable alternatives are implemented and shown to be effective. Agarwal et al. (2003) also observed that regular weekly administration was effective and is suitable for populations with mild to moderate anaemia. The results of Nguyen et al. (2008) also suggested for weekly IFA plus vitamin B-12 supplementation to Guatemalan women in reproductive age as efficacious as daily supplementation in improving serum folate status.

The results of the present study also indicate that there is remarkable improvement in the levels of Haemoglobin, even within 3 months of intervention period, especially among those from moderate to mild anaemia group. The additional advantages of ‘Weekly Iron Folic Acid Supplementation’ over ‘Daily Iron Folic Acid Supplementation’ are the ease of administration, cost effectiveness, better compliance to drug adherence, lesser adverse reactions and ease of monitoring and follow up actions among study subjects.

In a landmark study by Jayatissa et al. (1999) the prevalence of anaemia was reduced from 25% to 9.5% by weekly supplementation and from 18.5% to 8.6% by daily supplementation. The difference in mean rise of haemoglobin levels between the two groups receiving supplementation was not significant. On the basis of these results, they recommended long-term weekly doses of iron folate for the prevention of iron deficiency anaemia in adolescents. The use of the school as the administration channel also ensured better compliance in their study.

The present study underlines the findings of the Jayatissa et al. (1999) since here also the researchers have found overall improvement in anaemia status among the study subjects to the tune of 25% and 31.67% for daily and weekly regimes respectively.

Deshmukh et al. (2008) concluded, in their study conducted in Wardha (Maharashtra), that considering the biological and operational feasibility and the effectiveness of the intervention, weekly supplementation of iron to adolescent girls should be universally started in rural and tribal areas to correct the iron stores of women prior to becoming pregnant. Weekly iron folic acid supplementation combined with deworming every 6 months has been found as a feasible and cost effective intervention for the prevention of anaemia in adolescent girls in institutional and community settings by Vir et al. (2008) and they recommended that the weekly iron folic acid supplementation should therefore be made an integral part of education and reproductive health programs for achieving the Millennium Development Goals of improving maternal health and reducing child mortality. Garcia et al. (2005) in their 1998 Pangasinan pilot project also demonstrated weekly iron supplementation as an alternative to daily intake of iron supplements for the prevention of nutritional anaemia.

Hyder et al. (2002) observed in a comparative study that the compliance (ratio between observed and recommended tablet intake) was significantly higher in the weekly-supplementation regimen (93%) than in the daily-supplementation regimen (61%, p<0.05). Shah et al. (2002) also observed that dropouts because of poor compliance were almost double in the daily supplementation group as compared with the weekly group, and persistent adverse effects were also limited to the daily supplementation group. They reported weekly therapy to be equally effective yet causing fewer adverse effects, improves compliance, and reduces the cost of supplementation.

In the present study, we observed that the mean of unconsumed IFA was 6.1 + 10.98 in daily IFA group while it was 1.3 + 3.15 in weekly IFA group (p value by ‘t test’ = 0.001) underlining the observed better drug intake compliance among ‘Weekly Iron Folic Acid Supplementation’ group.

Patel et al. (2009) reported 63 % prevalence of anaemia amongst school children from Surat which decreased to 48.4% after one month of IFA supplementation given on alternate day. The overall increase in mean Hb was 0.59 gm% from 11.29 to 11.88. Kulkarni et al. (2012) found that the prevalence of anaemia was found to be very high (90.1%) among adolescent girls of same urban slum in Nagpur. Majority of the girls in this study were having mild or moderate anaemia (88.6%). Sharda et al. (2005) observed that only 29.43% girls were normal and 70.57% were affected with various grades of anaemic condition, 30.57% girls being mildly anaemic and 27.17% moderately anaemic while 12.83% suffered from severe anaemia among girls of scheduled caste community in Amritsar. Kaur et al. (2006), in their cross sectional study carried out in adolescent girls of four villages in rural Wardha, reported prevalence of anaemia as 59.8% which was significantly associated with low SES, low iron intake, vegetarian diet, worm infestation and history of excessive menstrual bleeding amongst the study population. Rawat et al. (2001), in their cross sectional study amongst 504 adolescent girls from rural area of Meerut (U.P), reported prevalence of anaemia to be 34.5% which was significantly associated with SES, type of family, family size, father’s occupation and mother’s education. Vasanthi et al. (1994) from NIN, Hyderabad, reported overall anaemia prevalence in 25% of girls irrespective of their urban rural residents and recommended the distribution of IFA tablets for correction of anaemia and iron deficiency in vulnerable groups.

While noting the varied prevalence rates of anaemia, as observed in studies cited above in various states of the country, it is worth emphasis that there may be regional differences in local strategies of anaemia control measures, but approach to the anaemia control through WIFS shall be more feasible, appropriate, resource friendly and result oriented.

## 5. Conclusion

The overall improvement in anaemia status among the study subjects is 25% and 31.67% for daily and weekly regimes respectively. Further the prevalence of Adverse Drug Reactions in daily regime is 13.3% as compared to 8.3% in weekly regime. The weekly regime shows abdominal pain as the only adverse drug reaction while for daily regime constipation, nausea, and skin rashes were also seen. The compliance to weekly regime, as evaluated from number of unconsumed ‘Iron Folic Acid’ capsules per study subject, is far better for weekly regime as compared to daily regime. This makes the weekly regime of ‘Iron Folic Acid’ supplementation as the promising tool for management of iron deficiency anaemia, particularly in adolescent girls. The present study, while observing the almost equal effectiveness of ‘Daily Iron Folic Acid Supplementation’ and ‘Weekly Iron Folic Acid Supplementation’ among adolescent girls, also strongly recommends the countrywide observance of the ‘Weekly Iron Folic Acid Supplementation’ regime mainly through school health programme, aanganwadi and other similar adolescent health care service providers, accredited social health activists, community physicians, social workers and community volunteers in India.
